# A Tarui Disease Phenotype with Compensated Hemolysis and a Homozygous *PFKM* Variant of Uncertain Significance Mimicking Chronic Myelomonocytic Leukemia

**DOI:** 10.3390/jcm15176924

**Published:** 2026-09-07

**Authors:** Andreea-Cornelia Neculcea, Ruxandra Aanicai, Barbara Massoto, Raluca Ileana Nistor, Carmen Fierbințeanu-Braticevici, Cristina Mambet, Alina Mititelu, Ana Maria Neagu, Cerasela Paraschiv, Mihai Popescu, Emilia Severin, Ana Maria Vlădăreanu

**Affiliations:** 1 Faculty of Medicine, “Carol Davila” University of Medicine and Pharmacy, 020027 Bucharest, Romania; andreea-cornelia.neculcea@drd.umfcd.ro (A.-C.N.); ileana.nistor@umfcd.ro (R.I.N.); carmen.fierbinteanu@umfcd.ro (C.F.-B.); cristina.mambet@umfcd.ro (C.M.); alina.mititelu@umfcd.ro (A.M.); ana.neagu@umfcd.ro (A.M.N.); cerasela.paraschiv@umfcd.ro (C.P.); mihai.popescu@umfcd.ro (M.P.); anamaria.vladareanu@umfcd.ro (A.M.V.); 2 Department of Hematology, Emergency University Hospital of Bucharest, 050098 Bucharest, Romania; 3 CENTOGENE GmbH, Am Strande 7, 18055 Rostock, Germany; ruxandra.aanicai@centogene.com (R.A.); barbara.massoto@centogene.com (B.M.); 4 Department of Neurology, Emergency University Hospital of Bucharest, 050098 Bucharest, Romania; 5 Department of Gastroenterology, Emergency University Hospital of Bucharest, 050098 Bucharest, Romania; 6 Department of Cellular and Molecular Pathology, “Stefan S. Nicolau” Institute of Virology, Romanian Academy, 030304 Bucharest, Romania; 7 Department of Intensive Care, Emergency University Hospital of Bucharest, 050098 Bucharest, Romania; 8 Department of Genetics, Faculty of Dentistry, “Carol Davila” University of Medicine and Pharmacy, 020027 Bucharest, Romania

**Keywords:** Tarui disease, glycogen storage disease type VII, *PFKM*, compensated hemolysis, rhabdomyolysis, chronic myelomonocytic leukemia

## Abstract

**Background and Clinical Significance**: Tarui disease, or glycogen storage disease type VII, is a rare autosomal recessive metabolic myopathy caused by muscle phosphofructokinase deficiency. Its manifestations include exercise intolerance, exertional myalgia, muscle cramps, myoglobinuria, rhabdomyolysis, hyperuricemia, and compensated hemolysis. The hematologic phenotype may obscure the underlying metabolic disorder and raise concern for a clonal myeloid neoplasm. **Case Presentation**: A 23-year-old man was referred for persistent mild thrombocytopenia following evaluation for jaundice and hepatosplenomegaly. He had undergone cholecystectomy at 18 years of age and reported exercise-induced myalgia, muscle cramps, and episodes of dark urine. Laboratory investigations demonstrated mild monocytosis, reticulocytosis, thrombocytopenia, hyperuricemia, elevated lactate dehydrogenase, and predominantly unconjugated hyperbilirubinemia, with a negative direct antiglobulin test. Selected inherited hemolytic disorders, hemoglobinopathies, and paroxysmal nocturnal hemoglobinuria were excluded. Bone marrow examination showed marked erythroid hyperplasia and mild megakaryocytic dysplasia. Testing for *JAK2*, *CALR*, and *MPL* mutations and an extended myeloid next-generation sequencing panel identified no pathogenic variants, and monocytosis resolved during follow-up. Whole-exome sequencing identified a homozygous *PFKM* missense variant, NM_001354735.1:c.1087A>T, p.(Ile363Phe), classified as a variant of uncertain significance. The patient subsequently developed severe rhabdomyolysis, with a creatine kinase level of 225,000 U/L and recovered after intensive intravenous hydration without renal impairment. **Conclusions**: Tarui disease should be considered in young patients with compensated hemolysis, hyperuricemia, exertional muscle symptoms, dark urine, or rhabdomyolysis, even when hematologic abnormalities suggest a myeloid disorder. The highly concordant phenotype and homozygous *PFKM* variant support a clinically probable diagnosis, although pathogenicity remains unconfirmed. Functional and segregation evidence may strengthen causal interpretation and support future variant reclassification.

## 1. Introduction

Chronic myelomonocytic leukemia (CMML) is a rare clonal hematopoietic malignancy classified as a myelodysplastic/myeloproliferative neoplasm (MDS/MPN) [1]. It is characterized by persistent peripheral blood monocytosis, bone marrow dysplasia, and an increased risk of progression to acute myeloid leukemia [2]. The diagnosis of CMML is based on the integration of clinical, morphologic, cytogenetic, and molecular findings, while excluding reactive causes of monocytosis and other myeloid neoplasms [3]. Diagnostic uncertainty may arise when monocytosis, cytopenias, splenomegaly, or bone marrow dysplasia occur in the context of a non-neoplastic disorder.

Tarui disease, also known as glycogen storage disease type VII, is a rare autosomal recessive disorder caused by biallelic variants in *PFKM*, which encodes the muscle isoform of phosphofructokinase [4]. PFK deficiency impairs glycolysis in skeletal muscle and erythrocytes [5]. The classical phenotype includes exercise intolerance, painful muscle cramps, exertional myalgia, episodic myoglobinuria, and rhabdomyolysis [6]. In addition, affected individuals may develop hyperuricemia and compensated hemolysis, characterized by reticulocytosis, indirect hyperbilirubinemia, and elevated lactate dehydrogenase despite normal or high-normal hemoglobin concentrations. Reduced erythrocyte 2,3-diphosphoglycerate concentrations may increase hemoglobin oxygen affinity and contribute to compensatory erythropoiesis, thereby masking anemia [7].

The coexistence of compensated hemolysis, hepatosplenomegaly, thrombocytopenia, mild monocytosis, and bone marrow abnormalities may prompt evaluation for a clonal hematologic disorder. However, exertional muscle symptoms, episodic dark urine, hyperuricemia, and rhabdomyolysis should raise suspicion of an underlying metabolic myopathy. We report a young man initially evaluated for CMML because of mild monocytosis, thrombocytopenia, hepatosplenomegaly, and marrow dysplasia, whose overall phenotype was highly suggestive of Tarui disease. Whole-exome sequencing identified a homozygous *PFKM* variant of uncertain significance that supported a clinically probable diagnosis in the context of a highly concordant phenotype.

## 2. Case Presentation

A 23-year-old male patient was referred to the Hematology Department for persistent mild thrombocytopenia, after one year of extensive diagnostic tests in the Gastroenterology Department. He had previously been diagnosed by a gastroenterologist with Gilbert syndrome. Many causes for hepatosplenomegaly were ruled out, including Wilson, Caroli, Gaucher, Niemann-Pick, Fabry, Pompe and Mucopolysaccharidoses type II, along with autoimmune hepatitis and myositis.

The patient was the sixth child in a family with no known history of anemia, hemolytic disorders, neuromuscular disease, or hereditary metabolic conditions. There was no history of consanguinity.

The patient recalled exercise-induced vomiting and severe headaches during school physical-education classes. By early adulthood, physical exertion consistently provoked myalgias and muscle cramps, followed by episodes of dark-colored urine. He had learned to avoid physical activity to prevent these symptoms, which substantially limited his daily functioning and ability to work.

Physical examination revealed moderate scleral and cutaneous jaundice, hepatosplenomegaly and an abdominal surgical scar from a cholecystectomy performed five years ago. Notably, the patient had undergone cholecystectomy at a relatively young age for presumed gallstone disease, although no medical records documenting the indication for surgery, stone confirmation, or stone type were available.

Neurologic evaluation was normal; the patient was alert and oriented, with no cranial nerve deficits, normal sensory examination, intact coordination, normal deep tendon reflexes, and normal gait. Brain computed tomography and subsequent magnetic resonance imaging showed no intracranial abnormalities. Electroencephalography was normal with no paroxysmal discharges. Nerve conduction studies and electromyography, including concentric needle examination of five muscles (anterior tibialis, vastus lateralis, extensor digitorum communis, biceps brachii, and deltoid), demonstrated normal insertional activity, no spontaneous rest activity, normal motor and sensory nerve conduction velocities, and normal interference pattern with recruitment.

At referral, the white blood cell count was 5.5 × 10^9^/L, with monocytes accounting for 11% of leukocytes and an absolute monocyte count of 0.605 × 10^9^/L. Hemoglobin was 16 g/dL, the reticulocyte count was 5.5%, and the platelet count was 130 × 10^9^/L. Biochemical testing showed hyperuricemia of 9 mg/dL, lactate dehydrogenase of 395 U/L (upper limit of normal, 221 U/L), and total bilirubin of 8 mg/dL, predominantly unconjugated. Abdominal computed tomography confirmed hepatosplenomegaly without other significant abnormalities.

Although the patient was not anemic, the history of cholecystectomy at a young age, along with reticulocytosis, elevated lactate dehydrogenase levels, and unconjugated hyperbilirubinemia raised suspicion of long-standing compensated chronic hemolysis. The direct antiglobulin test was negative. A comprehensive evaluation for inherited hemolytic disorders was performed. Glucose-6-phosphate dehydrogenase deficiency and pyruvate kinase deficiency were excluded, hemoglobin electrophoresis was unremarkable, and peripheral blood smear examination showed no significant red blood cell morphological abnormalities. The possibility of paroxysmal nocturnal hemoglobinuria (PNH) was excluded by flow cytometric analysis, which did not identify a PNH clone.

Biochemical and genetic investigations for porphyria were also performed. Urinary porphyrin analyses showed mild abnormalities, as summarized in Table 1.

An NGS-based gene panel with MLPA analysis for large deletions/duplications covering porphyria-associated genes (*ALAD*, *CPOX*, *FECH*, *HMBS*, *PPOX*, *UROD*, *UROS*) identified no pathogenic variants. A single heterozygous missense variant in *ALAD* (c.177G>C, p.[Lys59Asn]) was detected. The values of blood and urinary lead were normal.

Bone marrow aspirate revealed a hypercellular marrow with marked erythroid hyperplasia (56%) demonstrating a mixed normoblastic and megaloblastic maturation pattern and a reduced granulocyte-to-erythroid ratio (1:1). Granulocytic maturation was preserved (33%), and no increase in blasts was identified on morphologic assessment. Megakaryocytes were present, with both normal forms and small, hypolobulated, dysplastic megakaryocytes. Prussian blue staining showed sideroblasts at 3–4% without ringed sideroblasts. Bone marrow biopsy confirmed a hypercellular marrow with prominent erythroid hyperplasia in a paratrabecular distribution, consistent with a compensatory erythropoietic response. Immunohistochemistry demonstrated isolated CD34-positive cells at 2%, ruling out an increased blast population. Reticulin staining showed focal fibrosis (MF-1). Megakaryocytic dysplasia was again noted. The pathologist concluded that the findings were compatible with secondary erythroblastic hyperplasia, dyserythropoiesis and mild megakaryocytic dysplasia.

Molecular testing for myeloproliferative neoplasms was performed by RT-qPCR and targeted NGS. No mutations were identified in *JAK2* (exons 12–14, including V617F), *CALR* (exon 9), or *c-MPL* (exon 10). An extended 37-gene myeloid hot-spot NGS panel encompassing *TET2*, *SRSF2*, *ASXL1*, *CBL*, *DNMT3A*, *RUNX1*, *EZH2*, *NRAS*, *KRAS*, *NPM1*, *IDH1*, *IDH2*, *TP53*, *SF3B1*, *STAG2*, and others, with a reported analytical sensitivity of 99.8% for point mutations at >5% variant allele frequency, was also negative. Conventional cytogenetic analysis was unavailable.

At the 3-month hematologic reassessment, the white blood cell count was 5.1 × 10^9^/L, with monocytes accounting for 7% of leukocytes, corresponding to an absolute monocyte count of 0.357 × 10^9^/L. Hemoglobin and platelet counts remained stable.

The main clinical and diagnostic milestones are summarized in Table 2.

The timeline illustrates the transition from an initial evaluation for a possible clonal hematologic disorder to recognition of a combined hemolytic and metabolic myopathy phenotype. The diagnostic interpretation resulted from the integration of longitudinal hematologic findings, bone marrow morphology, molecular testing, clinical manifestations, and the subsequent episode of severe rhabdomyolysis. Abbreviations: CK, creatine kinase; G6PD, glucose-6-phosphate dehydrogenase; LDH, lactate dehydrogenase; NGS, next-generation sequencing; PNH, paroxysmal nocturnal hemoglobinuria; VUS, variant of uncertain significance; WBC, white blood cell count; WES, whole-exome sequencing.

Whole-exome sequencing was subsequently performed to investigate the underlying cause of the combined hemolytic and myopathic phenotype. WES identified a homozygous missense variant in *PFKM* (NM_001354735.1:c.1087A>T, p.[Ile363Phe]), classified as a variant of uncertain significance (VUS) according to ACMG recommendations (Table 3).

The variant was identified and reported based on WES data. Following re-evaluation, the reporting laboratory confirmed that the sequencing data were of sufficient quality and that orthogonal confirmation was not considered necessary.

Complete familial segregation analysis could not be performed because the mother was deceased and targeted testing of the father and siblings was not available. Enzymatic assessment of phosphofructokinase activity in erythrocytes or skeletal muscle was not available.

Additionally, WES identified two homozygous non-coding variants in *UGT1A1* (NM_000463.2.-3275T>G and c.-55_-54insAT), both classified as risk factors for Gilbert syndrome (OMIM #143500). These variants, with population allele frequencies of 0.55 and 0.35 in gnomAD, respectively, support the previous diagnosis of Gilbert syndrome. Serum bilirubin concentrations in Gilbert syndrome are typically between 1 and 5 mg/dL, although values approaching 6 mg/dL may occasionally occur, particularly during fasting, illness, dehydration, or physiological stress [8]. The total bilirubin concentration of 8 mg/dL observed in this patient is therefore higher than the range usually associated with isolated Gilbert syndrome.

Chronic hemolysis increases bilirubin production, whereas reduced UGT1A1 activity decreases hepatic bilirubin conjugation. Their coexistence may therefore produce more pronounced unconjugated hyperbilirubinemia than either condition alone. The marked unconjugated hyperbilirubinemia was therefore considered to result from a dual mechanism: increased bilirubin production due to chronic hemolysis, combined with reduced hepatic bilirubin conjugation associated with the homozygous *UGT1A1* risk variants. Gilbert syndrome contributed to the biochemical severity of the hyperbilirubinemia but did not explain the reticulocytosis, elevated lactate dehydrogenase, hepatosplenomegaly, or the broader myopathic phenotype.

The patient had one prior episode of rhabdomyolysis that was documented during the gastroenterological work-up, with a creatine kinase level of 6000 U/L. At the time of hematologic evaluation, basal creatine kinase was below 1000 U/L.

One month after the whole-exome sequencing result, the patient presented with severe bilateral arm pain, muscle cramps, hypotonia, and dark urine. The episode occurred in temporal association with sleep deprivation, carbohydrate intake (a bar of chocolate), and emotional stress. On admission, creatine kinase was 225,000 U/L, creatinine was 1 mg/dL, and aminotransferase concentrations were up to 50 times the upper limit of normal. Urinalysis obtained at the time of the acute presentation was unavailable because of the patient’s immediate transfer to the intensive care unit. In the intensive care unit creatine kinase decreased to 170,000 U/L within 24 h and further decreased daily; when the level of creatine kinase reached 20,000 U/L, the patient was transferred to the hematology department. Creatinine remained within the normal range throughout hospitalization, and electrolytes were consistently normal. Serum myoglobin was not available at the treating institution. Treatment consisted of intensive intravenous hydration in association with loop diuretics, prophylactic anticoagulation, folic acid, and allopurinol. Kidney function and urine output remained preserved, and renal replacement therapy was not required. He was discharged after seven days of supportive treatment, with creatine kinase declining to 1000 U/L and all other laboratory parameters normalized.

## 3. Discussion

### 3.1. Hematological Manifestations of Tarui Disease

This case shows the diagnostic challenges of Tarui disease. The combination of unconjugated hyperbilirubinemia, hepatosplenomegaly, reticulocytosis, elevated lactate dehydrogenase, early cholecystectomy, and hyperuricemia was thoroughly investigated by gastroenterologists and hematologists. Exercise intolerance, muscle pain and cramps, and recurrent dark urine after moderate physical activity suggested an underlying metabolic myopathy. Taken together, the combination of compensated hemolysis and exertional muscle symptoms strongly suggested phosphofructokinase deficiency [9].

The degree of hemolysis in Tarui disease may be underestimated because anemia can be absent despite shortened erythrocyte survival [7]. Phosphofructokinase-1 is a tetramer assembled from three tissue-associated subunit types encoded by *PFKM*, *PFKL*, and *PFKP*, commonly referred to as the muscle (M), liver (L), and platelet (P) isoforms, respectively. The composition of the tetramer varies among tissues. Skeletal muscle expresses predominantly the M_4_ homotetramer and is therefore highly dependent on the *PFKM*-encoded subunit. Human erythrocytes contain M- and L-type subunits assembled in different tetrameric combinations. Consequently, *PFKM* deficiency produces an almost complete loss of PFK activity in skeletal muscle but only a partial reduction in erythrocytes, where tetramers containing L subunits retain residual activity [5,10].

Reduced erythrocyte PFK activity limits glycolytic flux upstream of the formation of 1,3-bisphosphoglycerate, the precursor from which 2,3-diphosphoglycerate (2,3-DPG, also termed 2,3-bisphosphoglycerate) is generated through the Rapoport–Luebering shunt. The resulting decrease in erythrocyte 2,3-DPG increases hemoglobin–oxygen affinity and shifts the oxygen dissociation curve to the left, reducing oxygen release to peripheral tissues. The compensatory erythropoietic response may maintain a normal or high-normal hemoglobin concentration despite ongoing erythrocyte destruction [5,7]. This mechanism provides a plausible explanation for the combination of reticulocytosis, indirect hyperbilirubinemia, elevated lactate dehydrogenase, and normal baseline hemoglobin observed in our patient.

Another relevant clinical finding was the history of cholecystectomy at a young age. Even if the original indication could not be documented, pigment gallstones may complicate chronic hemolysis. Their presence at a young age should raise suspicion of an inherited red blood cell disorder. The lack of morphologic red blood cell abnormalities on peripheral smear further complicated the diagnostic process. Inherited causes of hemolysis, including glucose-6-phosphate dehydrogenase deficiency, pyruvate kinase deficiency, and hemoglobinopathies, were assessed. Paroxysmal nocturnal hemoglobinuria was also excluded by flow cytometry.

### 3.2. Tarui Disease in the Differential Diagnosis of CMML

Mild monocytosis, thrombocytopenia, splenomegaly, and bone marrow dysplasia initially raised concern for CMML or another myelodysplastic/myeloproliferative neoplasm. At referral, the absolute monocyte count was 0.605 × 10^9^/L, representing 11% of peripheral blood leukocytes. Although these values met the numerical thresholds for monocytosis used in current CMML classifications, they were documented at a single time point. CMML requires sustained absolute and relative peripheral blood monocytosis for more than three months, whereas reactive monocytosis associated with acute inflammation, tissue injury, physiological stress, or recovery from an acute illness is generally transient [1,2,3]. The isolated elevation observed in this patient therefore did not fulfill the persistence requirement for CMML.

The bone marrow findings also required interpretation in the context of the complete clinical and laboratory picture. Marked erythroid hyperplasia was consistent with a compensatory response to chronic hemolysis, whereas mild megakaryocytic dysplasia and focal MF-1 fibrosis were not, in isolation, sufficient to establish a clonal myeloid neoplasm. No increase in blasts was identified. Testing for *JAK2*, *CALR*, and *MPL*, as well as an extended 37-gene myeloid NGS panel, detected no pathogenic somatic variants. At the three-month reassessment, the absolute monocyte count had decreased to 0.357 × 10^9^/L, with monocytes representing 7% of leukocytes, confirming that persistent monocytosis was not present. This normalization supported a transient rather than sustained monocytosis. Although tissue injury associated with rhabdomyolysis can produce a reactive monocytic response, the available chronology and laboratory data did not allow the initial mild monocytosis to be attributed specifically to rhabdomyolysis.

Current diagnostic criteria for CMML require sustained peripheral blood monocytosis, with an absolute monocyte count ≥0.5 × 10^9^/L and monocytes accounting for ≥10% of leukocytes, together with compatible morphologic findings and exclusion of alternative causes [1,2]. In patients with an absolute monocyte count between 0.5 and 1.0 × 10^9^/L, supporting evidence of dysplasia and clonality assumes particular importance [2]. The diagnosis therefore requires integration of longitudinal blood counts, bone marrow morphology, cytogenetic and molecular findings, and exclusion of reactive causes of monocytosis [1,2,3].

Taken together, the resolution of monocytosis, absence of increased blasts or detectable myeloid-associated somatic variants, and predominance of compensatory erythroid hyperplasia reduced the likelihood of CMML or another MDS/MPN entity. Moreover, chronic compensated hemolysis, exertional myalgia, recurrent dark urine, hyperuricemia, and severe rhabdomyolysis were not explained by a clonal myeloid disorder and instead supported an underlying metabolic myopathy. Nevertheless, because conventional cytogenetic analysis was unavailable and the patient declined repeat bone marrow aspiration, a low-level or evolving clonal myeloid process could not be entirely excluded. Although its current probability is considered low, long-term hematologic follow-up is warranted, including serial complete blood counts and reassessment if persistent monocytosis, progressive cytopenias, circulating immature cells, or other findings suggestive of clonal evolution emerge.

### 3.3. Molecular Findings and Genotype–Phenotype Correlation

Although a heterozygous *ALAD* variant was identified during genetic testing, its clinical significance was questionable. The variant corresponds to the common ALAD*2 polymorphism, c.177G>C, p.(Lys59Asn), which has been associated with increased blood lead levels in environmentally exposed individuals, rather than with ALAD-deficiency porphyria [11]. Normal blood and urinary lead concentrations further argued against lead-related disease. The mild biochemical abnormalities in porphyrin metabolism were nonspecific and, in the absence of confirmatory biochemical and molecular evidence for porphyria, were not considered sufficient to explain the patient’s overall phenotype.

The homozygous *UGT1A1* risk variants supported the previous diagnosis of Gilbert syndrome but did not account for the broader hemolytic and myopathic phenotype.

Whole-exome sequencing identified a homozygous *PFKM* missense variant, NM_001354735.1:c.1087A>T, p.(Ile363Phe), classified by the reporting laboratory as a variant of uncertain significance according to ACMG/AMP criteria. The variant affected a highly conserved nucleotide, was absent from the population databases assessed by the laboratory and was predicted to be deleterious by multiple in silico tools. These findings support, but do not establish, pathogenicity. A previously reported homozygous *PFKM* missense variant, p.(Asp309Gly), was associated with a combined myopathic and hemolytic phenotype, markedly reduced erythrocyte PFK activity, and complete loss of muscle PFK activity. Structural modeling placed Asp309 near the allosteric ADP-binding site and suggested disruption of local hydrogen bonding [9]. This example supports the biological plausibility of a damaging *PFKM* missense substitution but does not establish the pathogenicity of p.(Ile363Phe), for which comparable functional or structural evidence is unavailable. In the context of the highly concordant phenotype, the homozygous p.(Ile363Phe) variant supports a clinically probable diagnosis of Tarui disease, although its pathogenicity remains unconfirmed. Measurement of PFK activity in erythrocytes would represent the least invasive functional investigation and is considered an appropriate method for confirming PFK deficiency when available [7,9]. A muscle biopsy could allow direct measurement of muscle PFK activity and histochemical assessment of glycogen accumulation but would be more invasive and should be considered only if the expected diagnostic benefit justifies the procedure. Markedly reduced PFK activity relative to laboratory-specific reference values and appropriately validated controls would support a damaging effect of the variant. However, no universally accepted residual-activity threshold specific to p.(Ile363Phe) has been established. Under the ACMG/AMP framework, the strength assigned to functional evidence depends on the validation, controls, reproducibility, and discriminatory capacity of the assay rather than on an arbitrary numerical cutoff [12,13]. Familial testing could provide complementary evidence. Demonstration that the father carries the variant in the heterozygous state would confirm paternal transmission. When both parents are available, demonstration of heterozygosity in each parent would confirm biparental inheritance. The strength of segregation evidence depends on the number of informative meioses, phenotype specificity, and the possibility of phenocopies rather than on a fixed requirement for two affected relatives [14]. Neither functional nor segregation evidence should be interpreted in isolation. Any future reclassification should integrate functional, segregation, population, computational, and phenotypic evidence within the ACMG/AMP framework.

### 3.4. Differential Diagnosis with Other Metabolic Myopathies

The exertional myalgia, muscle cramps, recurrent dark urine, and episodes of rhabdomyolysis also required consideration of other inherited metabolic myopathies. McArdle disease may present with exercise intolerance, painful contractures, and rhabdomyolysis and therefore represents an important clinical differential diagnosis. However, compensated hemolysis is characteristic of phosphofructokinase deficiency but is not a typical feature of McArdle disease. In addition, carbohydrate intake before exercise may improve exercise tolerance in McArdle disease, whereas carbohydrate administration may aggravate exercise intolerance in Tarui disease by increasing insulin-mediated glucose utilization and reducing the availability of alternative fuels [7].

Other considerations included phosphoglucomutase 1 deficiency, Pompe disease, fatty-acid oxidation disorders, and other inherited defects of glycolysis. Pompe disease had been investigated during the previous metabolic work-up and was not confirmed. PGM1 deficiency generally produces a broader multisystem phenotype that may include hepatic, endocrine, cardiac, and glycosylation abnormalities, which were not observed in this patient. Fatty-acid oxidation disorders are more commonly precipitated by prolonged exercise, fasting, intercurrent illness, or other catabolic stress. However, dedicated biochemical or functional testing did not definitively exclude all these disorders, and they were therefore regarded as less likely rather than formally excluded.

The combination of exertional muscle symptoms, severe rhabdomyolysis, chronic compensated hemolysis, hyperuricemia, and a homozygous *PFKM* variant provided the most coherent unifying explanation for the patient’s phenotype, while the absence of phosphofructokinase activity testing precluded definitive confirmation.

### 3.5. Rhabdomyolysis, Management, and Therapeutic Perspectives

The episode of rhabdomyolysis occurred in temporal association with sleep deprivation, carbohydrate intake, and emotional stress. The patient developed severe myalgia, cramps, hypotonia, dark urine, and a creatine kinase elevation to 225,000 U/L, consistent with severe rhabdomyolysis. Overall, the phenotype is most compatible with the classical myopathic form of GSD VII, accompanied by chronic compensated hemolysis. Although a predominantly hemolytic phenotype has been described in Tarui disease, the prominent exertional muscle symptoms and severe rhabdomyolysis in this patient support the classical myopathic presentation [7,15].

Carbohydrate intake before exercise may paradoxically worsen exercise tolerance in GSD VII, a response described as the “glucose paradox” or “out-of-wind” phenomenon [7]. Because the metabolic block is located at the phosphofructokinase step, the additional glucose cannot be efficiently used by PFK-deficient myocytes to sustain glycolytic ATP production. At the same time, the associated insulin response suppresses adipose-tissue lipolysis and hepatic ketogenesis, thereby reducing circulating free fatty acids and ketone bodies that could otherwise serve as alternative oxidative fuels during exercise [7,16]. This combination may intensify the muscle energy deficit and increase susceptibility to muscle injury and rhabdomyolysis. In this patient, chocolate intake may therefore have contributed to the episode. However, the exposure was reported in temporal association with the event, and no controlled metabolic assessment was performed; a direct causal relationship cannot be established. Sleep deprivation and emotional stress are not recognized as disease-specific triggers of GSD VII, although they may have acted as nonspecific physiological stressors.

No established disease-modifying treatment is currently available for Tarui disease. Long-term management is individualized and focuses primarily on preventing muscle injury and recognizing rhabdomyolysis at an early stage. Patients should avoid high-intensity anaerobic or isometric exercise, prolonged fasting, dehydration, and large carbohydrate loads immediately before physical activity. New or unusually severe myalgia, muscle weakness, or dark urine should prompt cessation of physical activity, adequate hydration, and early medical assessment. Following the episode, the patient received dietary and lifestyle counseling consistent with these preventive principles. Physical activity should not necessarily be eliminated but should be adapted to individual tolerance, preferably with guidance from clinicians experienced in metabolic myopathies [7].

Long-term follow-up may include periodic clinical assessment and monitoring of creatine kinase, renal function, serum electrolytes, uric acid, hematologic parameters, and markers of hemolysis, according to the clinical course. Nutritional counseling and coordinated follow-up involving metabolic, neurologic, hematologic, and genetic expertise are advisable. A ketogenic diet has been associated with symptomatic improvement in a single-patient report and should be considered only under the supervision of a metabolic physician and dietitian [16]. Mitapivat has shown promising results in one patient with genetically confirmed phosphofructokinase deficiency, including improvement in exercise tolerance, muscle symptoms, selected hemolysis parameters, and creatine kinase levels [17]. However, the evidence for both approaches remains limited to individual reports, and mitapivat should be regarded as an experimental, off-label option requiring specialist supervision.

### 3.6. Limitations

This case has several limitations. Conventional cytogenetic analysis was not performed, and the patient declined repeat bone marrow examination. Therefore, although the resolution of monocytosis and the absence of detectable variants on the myeloid NGS panel substantially reduced the likelihood of CMML or another MDS/MPN entity, a low-level or evolving clonal myeloid process cannot be excluded with absolute certainty. Continued hematologic surveillance is therefore warranted.

PFK activity could not be measured in erythrocytes or skeletal muscle, and a muscle biopsy was not performed. Familial segregation analysis could not be completed because the patient’s mother was deceased and targeted testing of the father and siblings was not available. A validated erythrocyte or muscle PFK assay demonstrating markedly reduced activity would provide relevant functional evidence, while testing informative relatives could support biparental inheritance and co-segregation. However, neither an isolated enzyme result nor a predetermined number of affected relatives would automatically justify variant reclassification. The strength of such evidence would need to be evaluated within the ACMG/AMP framework [12,13,14].

The homozygous *PFKM* variant therefore remains classified as a variant of uncertain significance despite its rarity, homozygous state, high nucleotide conservation, deleterious in silico predictions, and strong phenotypic concordance. Functional and familial evidence, together with future clinical and population data, may strengthen its causal interpretation and support reassessment as additional evidence becomes available.

This report highlights a clinically probable Tarui disease associated with a homozygous *PFKM* variant of uncertain significance investigated from a hematology perspective, in which initial concern for CMML prompted extensive diagnostic evaluation. In patients with compensated hemolysis, hyperuricemia, exercise-induced myalgia, intermittent dark urine episodes, and rhabdomyolysis, Tarui disease should be included in the differential diagnosis to avoid misclassification as a hematologic malignancy and unnecessary treatment.

## 4. Conclusions

From a hematological standpoint, unexplained compensated hemolysis combined with exertional myalgia, dark urine, or rhabdomyolysis should prompt evaluation for glycolytic enzyme defects or other metabolic myopathies, even when monocytosis, cytopenia, splenomegaly, or bone marrow dysplasia raise concern for a clonal myeloid disorder. In the presence of a highly concordant clinical phenotype, a homozygous *PFKM* variant may guide the diagnostic pathway. Functional evidence, such as reduced PFK activity, together with familial segregation data, would strengthen the causal interpretation of the variant and may support its future reclassification.

## Figures and Tables

**Table 1:** Urinary porphyrin analyses.

Porphyria Tests	Results	Normal Range
Total porphyrins	254.562 µg/24 h	<220 µg/24 h
Uroporphyrins	84.467 µg/24 h	15–50 µg/24 h
Coproporphyrins	170.095 µg/24 h	35–150 µg/24 h
Delta aminolevulinic acid	5.632 mg/24 h	<6.43 mg/24 h
Porphobilinogen in urine	2.794 mg/24 h	<1.7 mg/24 h

**Table 2:** Timeline of the main clinical and diagnostic milestones.

Age/Time Point	Clinical Event or Finding	Main Investigations/Results	Diagnostic Significance
Adolescence	Exercise-induced vomiting and severe headaches during physical-education classes	No specific investigations documented	Early exertional symptoms suggestive of an underlying metabolic myopathy
Early adulthood	Recurrent exertional myalgia, muscle cramps, and episodes of dark urine	Symptoms led the patient to avoid physical activity	Increased suspicion of exercise-induced muscle energy impairment
18 years	Cholecystectomy for presumed gallstone disease	Operative records and stone composition unavailable	Possible indirect consequence of chronic hemolysis
Approximately 22 years	Gastroenterological evaluation for jaundice and hepatosplenomegaly	Predominantly unconjugated hyperbilirubinemia; diagnosis of Gilbert syndrome; multiple hepatic and metabolic disorders excluded	Persistent jaundice remained incompletely explained
During gastroenterological work-up	First documented episode of rhabdomyolysis	Creatine kinase approximately 6000 U/L	Supported the presence of a metabolic myopathy
23 years—hematology referral	Mild thrombocytopenia, reticulocytosis, mild monocytosis, hepatosplenomegaly, and compensated hemolysis	WBC 5.5 × 10^9^/L; monocytes 11%, absolute monocyte counts 0.605 × 10^9^/L; hemoglobin 16 g/dL; reticulocytes 5.5%; platelets 130 × 10^9^/L; LDH 395 U/L; total bilirubin 8 mg/dL; uric acid 9 mg/dL	Raised concern for a clonal myeloid disorder but also supported chronic compensated hemolysis
Initial hematologic work-up	Investigation of hemolysis and porphyria	Direct antiglobulin test negative; G6PD deficiency, pyruvate kinase deficiency, hemoglobinopathies, and PNH excluded; porphyrin abnormalities nonspecific	Common causes of hemolysis and porphyria were not confirmed
Bone marrow evaluation	Hypercellular marrow with marked erythroid hyperplasia and mild dysplastic changes	Erythroid precursors 56%; no blast increase; CD34-positive cells approximately 2%; MF-1 fibrosis; mild megakaryocytic dysplasia	Findings favored secondary erythroid hyperplasia but prompted assessment for a myeloid neoplasm
Molecular hematology work-up	Evaluation for clonal myeloid disease	*JAK2*, *CALR*, and *MPL* negative; 37-gene myeloid NGS panel negative; conventional cytogenetics unavailable	Reduced the likelihood of a clonal myeloid neoplasm
Three-month follow-up	Resolution of monocytosis	WBC 5.1 × 10^9^/L; monocytes 7%; absolute monocyte count 0.357 × 10^9^/L	Persistent monocytosis required for CMML was not demonstrated
Subsequent whole-exome sequencing	Identification of a candidate molecular finding	Homozygous *PFKM* c.1087A>T, p.(Ile363Phe), classified as VUS; two homozygous *UGT1A1* risk variants also identified	Supported a clinically probable diagnosis of Tarui disease and confirmed a genetic background compatible with Gilbert syndrome
One month after WES	Severe rhabdomyolysis	CK 225,000 U/L; creatinine 1 mg/dL; marked aminotransferase elevation; electrolytes preserved	Strong phenotypic evidence for the classical myopathic form of GSD VII
Hospital course	Supportive treatment	Intensive intravenous hydration, loop diuretics, prophylactic anticoagulation, folic acid, and allopurinol; CK decreased to 1000 U/L at discharge	Recovery without renal impairment
Current interpretation	Clinically probable Tarui disease	Segregation analysis and PFK activity testing unavailable; variant remains VUS	Functional and familial evidence would strengthen causal interpretation and support possible reclassification

**Table 3:** Molecular characteristics of the identified *PFKM* variant.

Parameter	Result
Gene	*PFKM*
Transcript	NM_001354735.1
cDNA variant	c.1087A>T
Predicted protein consequence	p.(Ile363Phe)
Zygosity	Homozygous
Variant type	Missense
SNP identifier	Not available
PolyPhen-2	Probably damaging
SIFT	Deleterious
MutationTaster	Disease-causing
Nucleotide conservation	High
Population databases assessed	Absent from gnomAD, ESP, and 1000 Genomes
Laboratory classification	Variant of uncertain significance, according to ACMG recommendations

## Data Availability

All data supporting the findings of this case report are included within the article.
